# Psychosocial health disparities in early childhood: Socioeconomic status and parent migration background

**DOI:** 10.1016/j.ssmph.2022.101137

**Published:** 2022-06-06

**Authors:** Jie Luo, Amy van Grieken, Junwen Yang-Huang, Suzanne J. van den Toren, Hein Raat

**Affiliations:** Erasmus MC, Department of Public Health, Rotterdam, the Netherlands

**Keywords:** Psychosocial problems, Socio-emotional development, Socioeconomic status, Health disparity, Ethnicity, Preschool children

## Abstract

The association between low socioeconomic status (SES), migration background and psychosocial health could be various in different age stages, rare research has investigated associations in very early childhood. Cross-sectional data of SES, parental migration background, and child’s psychosocial problems among 2149 children were collected (*M*_age_ = 24.6 ± 1.8 months, 49.9% girls) from a community population. Indicators of SES included parental education level, maternal work status, and family composition. Child’s psychosocial problems, including social-emotional problems and delay in social-emotional competence, were assessed by the Brief Infant–Toddler Social and Emotional Assessment Problem scale and Competence scale, respectively. Interaction effects between SES and maternal migration background in risk of psychosocial problems were found. Among children of a native-born mother, lower maternal and paternal education levels indicated a higher risk of social-emotional problems and competence delay, respectively. Children of a migrant mother had a higher risk of both social-emotional problems and competence delay if they had a migrant father. The results highlight psychosocial health disparities in 2-year-old children and the need for research into mechanisms underlying these associations.

## Highlights

• Psychosocial problems, including social-emotional problems and delay in social-emotional competence, in 2-year-old sample were evaluated.
• Interaction between maternal education level and maternal migration background was observed in risk of psychosocial problems.
• Among children of a native-born mother, lower maternal and paternal education levels indicate a higher risk of psychosocial problems.
• Among children of a migrant mother, there was a higher risk of psychosocial problems if they had a migrant father.

## 1 Introduction

Psychosocial health consists of emotional, behavioral and social well-being. The most apparent psychosocial health problems in school-age children and adolescents are depression, anxiety, hyperactivity, and difficulties in social interaction (Klitzing et al., 2015). In the first three years of life, the main types of problems in psychosocial competence are presented as indifference, noncompliance or anti-social behavior (Briggs-Gowan et al., 2004). Although psychosocial problems are estimated at 10–20% among children and adolescents worldwide (WHO, 2021), problems often go overlooked especially in preschool children (Lyons-Ruth et al., 2017). Psychosocial problems in early childhood could track into adulthood and indicate a higher risk of substance use, depression, anxiety, and disability (Arslan et al., 2021; O'Connor et al., 2018; WHO, 2021).

Individual, economic and social factors are known to be associated with psychosocial health (WHO, 2021). Socioeconomic status (SES), may be particularly important for children’s psychosocial health due to the impact of the family situation on child development (Kuruczova et al., 2020; Mullola et al., 2021). Growing up in a context of socioeconomic disadvantages is associated with a higher risk of psychosocial problems (Collishaw et al., 2019; Herrmann et al., 2018; Kirby et al., 2020). Socioeconomic status as a multidimensional concept is most frequently assessed by a series of indicators, including family income, education level, and work status (Allen et al., 2014). Currently, in child’s psychosocial health studies, studies on the association between indicators of SES and psychosocial health among children show inconsistent results. Some studies (Collishaw et al., 2019; Herrmann et al., 2018) suggest that lower SES, as indicated by family income, parental education and work status, is associated with worse psychosocial health. Meanwhile others (Reiss et al., 2019; Siponen et al., 2011) found no association using similar indicators of SES and children's mental health. Therefore, it has been suggested that assessing a broad range of indicators of SES (e.g. family composition) offers a more comprehensive view of socioeconomic inequalities in children's psychosocial health problems (Kuruczova et al., 2020).

Furthermore, migration background has been reported to contribute to poor mental health (Kim et al., 2020; Mock-Muñoz de Luna et al., 2019). The review by Mock-Muñoz de Luna et al. reported that non-western migrant children had a higher prevalence of mental and physical problems compared to the majority of children in Scandinavian countries, and some of these differences remained significant after adjusting for SES(Mock-Muñoz de Luna et al., 2019). Ethnic health inequalities were also observed in a study in the Netherlands among 5-12-year-old children (Crone et al., 2010). Crone et al. reported that children with a migration background (i.e.a Moroccan, Turkish, Surinam, or Antillean background) had higher total and internalizing problem scores as measured by the Child Behavior Checklist (CBCL)(Crone et al., 2010). The subgroups of immigrants in studies are diverse, including highly skilled migrants, but also refugees, asylum seekers, and/or unskilled labor workers who tended to have poor physical and mental health (Kim et al., 2020; Mock-Muñoz de Luna et al., 2019). The cultural background of migrants has been considered to be a barrier in health care access; for example, migrant parents from non-western countries may discuss problems and concerns mostly within the family circle (Crone et al., 2010).

Additionally, even though socioeconomic disparities in mental health may occur in all age groups, the impact of low SES on mental health may be stronger in early childhood than in adolescence (Reiss, 2013). However, most association studies in this area (Timalsina et al., 2018; Kim et al., 2020; Collishaw et al., 2019; Behere et al., 2017; Siponen et al., 2011) are conducted among older children (age>10 years). Evidence on the association between SES and psychosocial problems in preschool children is relatively scarce (Poulain et al., 2020).

This study aims to explore associations between indicators of SES (i.e. parental education level, maternal work status, and family composition), parental migration background and children’s psychosocial problems among a large population-based sample of 2-year-old children. We hypothesized that children of parents with a lower education level and a migration background have a higher risk of psychosocial problems, compared to children of parents with a high education level and a native-born background.

## 2 Methods

### 2.1 Study design and population

A cross-sectional study design was applied using data from a cohort study. In 2014 and 2015, Dutch Preventive Youth Health Care (YHC) invited parents living in the Rotterdam–Rijnmond area to participate in the study accompanying the regular well-child visit invitation at children age 2 years. An invitation letter accompanied by a leaflet and letter to introduce the study, a consent form and the baseline questionnaire were sent to parents. In total, 2316 parents consented to participate in the study and 2305 parents completed the questionnaires (response rate 99.5%). Twins in this study shared the same socioeconomic status and migration background; therefore, only one of the two children from twins was included in the population for analyses (n = 31 children were excluded). The parental education level and work status were assessed for the respondent and their partner’s situation. Therefore, questionnaires filled by other caregivers than parents (e.g. grandparents, siblings, and other relatives of the child) were excluded (n = 55). And children with missing on psychosocial problems were excluded (n = 70). Thus, 2149 children (*M*_age_ = 24.6 ± 1.8 months, 50.1% boys) were included in the analyses of this study (see Supplementary Fig. 1).

Post hoc power analyses were performed, based on the different proportions of children with psychosocial problems (i.e., social-emotional problems and competence delay) between subgroups of indicators of SES and parental migration background (IBM, 2022). The defaulted Test Method (Chi-square test) and Estimation Method (Normal approximation) were used in the power calculation with a significance level of 0.05. The minimum power in the current study was 0.94 for the outcome of social-emotional problems and 0.80 for the outcome of competence delay in detecting the different proportions between subgroups.

### 2.2 Indicators of socioeconomic status

The education level of both parents, maternal work status, and family composition were assessed as indicators of SES. The education level of both parents was categorized as high (higher vocational education, university), middle (higher secondary education, vocational education) or low (primary education, lower secondary education)(CBS, 2018b).

Respondents to the questionnaire were asked to report their work status. Therefore, 88.5% reflects the maternal work status and 11.5% paternal work status. Work status was categorized into three categories: full-time, part-time, and unemployed. If a questionnaire was completed by a father, then the value of work status would be regarded as missing. The family composition was classified as: ‘two-parent family’ and ‘single-parent family’.

### 2.3 Migration background

Migration background of both parents was defined by the country of birth of the child's grandparents on each side according to the Classification of Statistics Netherlands (CBS, 2018a). When with regard to one parent, both grandparents were born in the Netherlands, the parent was considered to be native-born. When either one grandparents were born outside the Netherlands, the parent was considered to have a migration background. When both grandparents were born outside the Netherlands, the parent's migration background was decided by the grandmother's land of birth (CBS, 2018a). The binary migration background variable (Native-born vs migrant) was used in statistical analyses in order to have enough statistical power. Parents with a Dutch background were regarded as native-born parents; parents with a western (excl. the Netherlands) or non-western background were regarded as migrant parents according to the classification of Statistics Netherlands (CBS, 2018a). The frequency and proportion of parents with a migrant background (western vs non-western) are presented in the Supplementary Table S1.

### 2.4 Psychosocial problems

Psychosocial problems in this study were characterized by social-emotional problems and delay in social-emotional competence, measured by the Brief Infant–Toddler Social and Emotional Assessment (BITSEA)(Briggs-Gowan et al., 2004). The BITSEA is a 42-item questionnaire which consists of a 31-item Problem scale and an 11-item Competence scale. Each item is scored 0 for ‘not true’, 1 for ‘somewhat true’, and 2 for ‘certainly true’ (Briggs-Gowan et al., 2004). The items from the two scales of the BITSEA are summed up independently. A score ≥14 on the Problem scale is categorized as ‘at risk of psychosocial problems’, and a score of ≤15 on the Competence scale is categorized as ‘at risk of competence delay’ (Kruizinga et al., 2012). The BITSEA is specifically designed for screening the social-emotional problems (e.g., anxiety, depression or anti-social behavior) by Problem scale and competence delay (e.g., indifference, noncompliance or dysregulation) by Competence scale in children aged 12–36 months (Kruizinga et al., 2012; Briggs-Gowan et al., 2004). Of the children at risk of psychosocial problems and with competence delay at 12- to 36-month-olds as assessed by BITSEA, 42.5% and 43.8% were at increased risk for psychiatric disorders at age six measured by the Diagnostic Interview Schedule for Children (DISC)(Briggs-Gowan & Carter, 2008). The Cronbach’s alphas in this study are 0.77 and 0.55.

### 2.5 Potential covariates

Potential confounding factors were chosen by biological plausibility and data availability (Reiss et al., 2019). Child’s gender, child’s age, gender and age of the respondents of questionnaire, and previous help-seeking for psychosocial problem (yes/no) were self-reported. Previous help-seeking for psychosocial problems was measured by the question: ‘‘Have you sought help for your child to support his/her social-emotional development from the following sources in the past two years?'. The responses were dichotomized into ‘no’ (no confirmatory responses) and ’yes’ (one or more confirmatory responses).

### 2.6 Statistical analyses

Descriptive statistics were applied to describe family characteristics and child characteristics. Differences between children with and without risk of psychosocial problems with regard to socio-demographic characteristics were tested by T-tests for continuous variables and Chi-square tests for categorical variables. Effect sizes were measured by Cramer's V for the chi-square test and Cohen’s d for T-tests. Multiple comparisons were conducted among 3-category-variables by Bonferroni adjusted z-tests. Logistic regression models were applied to study the associations between indicators of SES and risk of psychosocial problems. Covariates for adjusting models were selected by testing the univariate association using Chi-square tests and T-tests. Only those factors significantly, p < 0.05, associated with the outcome (either socio-emotional problems, competence delay or both) were added to the models as covariates. In the present study, only child’s gender and previous help-seeking for psychosocial problems were associated with children’s psychosocial problems in the univariate logistic regression (p < 0.05; data not shown). Therefore, all the models were adjusted for child’s gender and previous help-seeking for psychosocial problems.

**Fig. 1:**
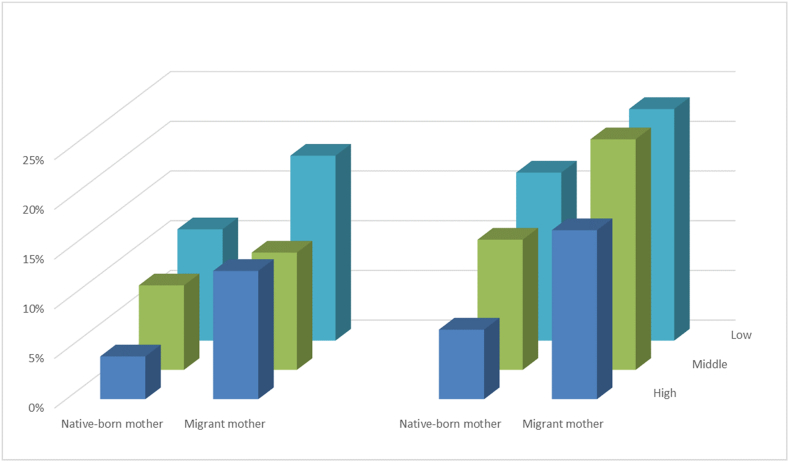
Prevalence of social-emotional problems (left) and competence delay (right) in subgroups of maternal education level and maternal migration background.

Interaction terms between different indicator of SES and parental migration background were added to the models and considered statistically significant at p < 0.05. A significant interaction was observed between maternal education level and maternal migration background in the model for social-emotional problems (*p* = 0.031), and in the model for competence delay (*p* = 0.024). Maternal migration background (native-born vs. migrant) modified the association between risk of social-emotional problems as well as competence delay. Therefore, the results are presented for children from native-born mothers and migrant mothers separately. Fig. 1 showed prevalence of social-emotional problems and prevalence of competence delay among children by maternal education level in subgroups of native-born mothers and migrant mothers.

Multiple imputation by Fully Conditional Specification was applied to deal with missing values in independent variables in SPSS. Maternal work status was the variable with the maximum missing cases (n = 329, 15.3%) among the sample of 2149 parents. The pooled results of five imputed datasets were used to report odds ratios (ORs), and their 95% confidence intervals (CIs). Excluded children (n = 156) were compared to children from included participants (n = 2149) in this study to check the selection bias. The robustness of results and potential bias introduced by multiple imputation was checked in two ways: 1) sensitivity analysis using the non-imputed complete-case data; 2) sensitivity analysis using data with missing values in maternal work status were all imputed as full-time, part-time and unemployed, respectively. All analyses were completed using IBM SPSS version 25 for Windows (IBM Corp., Armonk, NY, USA). A p-value of <0.05 was considered to be statistically significant.

## 3 Results

### 3.1 Characteristics of the study population

Table 1 presents the characteristics of the study population by risk of social-emotional problems and by risk of competence delay separately. Among the 2149 children, 188 (8.7%) were at risk of social-emotional problems and 297 (13.8%) were at risk of competence delay. Child’s mean age was 24.6 (SD = 1.8) months and the half (49.9%) were girls. The mean age of parents was 33.2 (SD = 5.3) years. Supplementary Table S1 shows circa 70% of migrant parents (mother 69.4%; father 70.6%) had a non-western background, and children of parents with a non-western background were more likely to be at risk of social-emotional/behavioral problems compared to their peers of parents with a western background. Compared to parents of children without any risk of psychosocial problems, both parents of children at risk more often had a lower education level and a migration background, were more often unemployed, and more often formed a single-parent household (all *p* values < 0.05).

**Table 1:** Sociodemographic characteristics of the study population at child age 2-years by social-emotional problems and competence delay (N = 2149).

	**Total (n=2149)**	Children at risk of social-emotional problems		*t/*χ2	*P* value	Effect size	Children at risk of competence delay		*t/*χ2	*P* value	Effect size
		No (n = 1961)	Yes (n = 188)				No (n = 1852)	Yes (n = 297)			
***Family characteristics***											
**Parental age in years**	33.2 ± 5.3	33.2 ± 5.3	32.4 ± 5.7	1.97	0.050	0.16	33.1 ± 5.2	33.8 ± 6.1	−1.94	0.054	−0.14
**Respondent of questionnaire**				1.11	0.293	0.02			5.41	**0.020**	0.05
**Mother**	1902 (88.5)	1740 (88.7)	162 (86.2)				1651 (89.1)	251 (84.5)			
**Father**	247 (11.5)	221 (11.3)	26 (13.8)				201 (10.9)	46 (15.5)			
**Maternal education level**				21.44	**<0.001**	0.10			26.05	**<0.001**	0.11
**High**	1113 (53.2)	1040 (54.4) *	73 (40.6)*				1001 (55.4)*	112 (39.6)*			
**Middle**	796 (38.1)	719 (37.6)	77 (42.8)				662 (36.6)*	134 (47.3)*			
**Low**	182 (8.7)	152 (8.0)*	30 (16.7) *				145 (8.0)*	37 (13.1)*			
**Paternal education level**				37.55	**<0.001**	0.14			10.38	**0.006**	0.07
**High**	960 (47.5)	915 (49.4)*	45 (26.6)*				853 (48.7)*	107 (39.8)*			
**Middle**	788 (39.0)	705 (38.1)*	83 (49.1)*				676 (38.6)	112 (41.6)			
**Low**	273 (13.5)	232 (12.5)*	41 (24.3)*				232 (12.7)*	50 (18.6)*			
**Maternal work status**				21.35	**<0.001**	0.11			20.22	**<0.001**	0.11
**Full-time**	261 (14.3)	247 (14.8)*	14 (9.0)*				228 (14.3)	33 (14.3)			
**Part-time**	1136 (62.4)	1053 (63.3)*	83 (53.2)*				1018 (64.1)*	118 (51.1)*			
**Unemployed**	423 (23.2)	364 (21.9)*	59 (37.8)*				343 (21.6)*	80 (34.6)*			
**Maternal migrant background**				24.97	**<0.001**	0.11			49.58	**<0.001**	0.16
**Native-born**	1401 (67.8)	1310 (69.4)	91 (51.1)				1264 (70.7)	137 (49.5)			
**Migrant**	664 (32.2)	577 (30.6)	87 (48.9)				524 (29.3)	140 (50.5)			
**Paternal migrant background**				29.89	**<0.001**	0.12			34.58	**<0.001**	0.13
**Native-born**	1456 (70.9)	1364 (72.6)	92 (52.9)				1301 (73.2)	155 (56.0)			
**Migrant**	598 (29.1)	516 (27.4)	82 (47.1)				476 (26.8)	122 (44.0)			
**Family composition**				19.28	**<0.001**	0.10			9.39	**0.002**	0.07
**Two-parent**	1916 (91.5)	1768 (92.3)	148 (82.7)				1667 (92.2)	249 (86.8)			
**Single-parent**	179 (8.5)	148 (7.7)	31 (17.3)				141 (7.8)	38 (13.2)			
***Child characteristics***											
**Age in months**	24.6 ± 1.8	24.6 ± 1.8	24.6 ± 1.6	−0.46	0.644	−0.04	24.6 ± 1.8	24.4 ± 1.8	1.92	0.055	0.12
**Gender**				13.58	**<0.001**	0.08			30.65	**<0.001**	0.12
**Girl**	1072 (49.9)	1002 (51.3)	70 (37.2)				968 (52.5)	104 (35.1)			
**Boy**	1069 (50.1)	951 (48.7)	118 (62.8)				877 (47.5)	192 (64.9)			
**Previous help seeking**				92.77	**<0.001**	0.21			2.45	0.117	0.03
**No**	1656 (79.5)	1566 (82.1)	90 (51.4)				1440 (80.1)	216 (76.1)			
**Yes**	426 (20.5)	341 (17.9)	85 (48.6)				358 (19.9)	68 (23.9)			

P values were based on independent T test and χ2 tests. Significant p values are presented in bold.

*Significant difference between two subgroups at 0.05 level in multiple comparison by Bonferroni adjusted z-tests for column proportions. Data presented as mean ± SD or number (percentage).

Number of missing: Parental age = 31; Maternal education level = 58; Paternal education level = 128; Maternal work status = 329; Maternal migrant background = 84; Paternal migrant background = 95; Family composition = 54; Child age = 15; Child gender = 8; Previous help seeking = 67.

### 3.2 Association between SES and children’s psychosocial problems

Among children of a native-born mother, 6.5% and 9.8% of the children were at risk of social-emotional problems or at risk of competence delay; and these percentages were 13.1% and 21.1% among children of a migrant mother. Children of a migrant mother had a higher risk of both social-emotional problems and competence delay compared to their peers of a native-born mother (*p* < 0.001, data not shown).

Table 2 presents the association between SES indicators and risk of psychosocial problems in subgroups of children of a native-born mother and a migrant mother. In the subgroup of native-born mothers, children of a low-educated (OR = 3.71, 95%CI:1.24–5.96) or a middle-educated father (OR = 2.38, 95%CI:1.32–4.31) had a higher risk of social-emotional problems, compared to children of a high-educated father. Children of a low-educated (OR = 1.54, 95%CI:1.23–5.25) or middle-educated (OR = 1.86, 95%CI:1.24–2.78) mother had a higher risk of competence delay, compared to children of a high-educated mother. In the subgroup of migrant mothers, a higher risk of both social-emotional problems (OR = 2.19, 95%CI:1.21–3.96) and competence delay (OR = 1.79, 95%CI:1.06–3.02) was observed for children of a migrant father, compared to children of a native-born father. No significant associations were observed for maternal work status and family composition (*p* > 0.05).

**Table 2:** Association between indicators of SES, parental migration background and social-emotional problems (N = 2149).

	Subgroup of native-born mothers		Subgroup of migrant mothers	
	Children at risk of social-emotional problems	Children at risk of competence delay	Children at risk of social-emotional problems	Children at risk of competence delay
	OR (95%CI)	OR (95%CI)	OR (95%CI)	OR (95%CI)
**Maternal education level**				
**High**	Ref	Ref	Ref	Ref
**Middle**	1.24 (0.73–2.12)	1.86 (1.24–2.78)*	0.56 (0.31–1.01)	1.42 (0.85–2.36)
**Low**	1.21 (0.46–3.14)	1.54 (1.23–5.25)*	1.13 (0.52–2.49)	1.12 (0.56–2.23)
**Paternal education level**				
**High**	Ref	Ref	Ref	Ref
**Middle**	2.38 (1.32–4.31)*	1.05 (0.69–1.61)	1.83 (0.97–3.43)	0.92 (0.57–1.49)
**Low**	3.71 (1.24–5.96)*	0.71 (0.35–1.47)	1.68 (0.76–3.75)	1.37 (0.75–2.50)
**Maternal work status**				
**Full-time**	Ref	Ref	Ref	Ref
**Part-time**	3.00 (0.89–10.03)	0.93 (0.51–1.70)	1.51 (0.72–3.19)	0.97 (0.38–2.40)
**Unemployed**	3.25 (0.83–12.63)	1.32 (0.68–2.60)	2.02 (0.97–4.23)	1.42 (0.75–2.70)
**Paternal migrant background**				
**Native-born**	Ref	Ref	Ref	Ref
**Migrant**	1.13 (0.57–2.23)	1.00 (0.55–1.83)	2.19 (1.21–3.96)*	1.79 (1.06–3.02)*
**Family composition**				
**Two-parent**	Ref	Ref	Ref	Ref
**Single-parent**	1.82 (0.87–3.81)	1.66 (0.86–3.19)	1.14 (0.63–2.04)	0.79 (0.46–1.35)

Abbreviation: OR = odds ratio; CI = confidence internal. The analyses were conducted on imputed data.

The model has been adjusted for covariates: child gender and previous help seeking.

*p < 0.05.

### 3.3 Sensitivity analyses

Supplementary Table S2 shows the results of logistic regression conducted with complete data. There was one difference between the multivariable logistic regression model conducted with non-imputed complete-case data and the model with imputed data regarding significance level of paternal migration background. In the imputed data analysis, children of a migrant father had a significant higher risk of social-emotional problems (OR = 2.19, 95%CI:1.21–3.96). This association was comparable but not significant in the analysis conducted with non-imputed data (OR = 2.02, 95%CI: 0.98–4.17). Although the significance of this factor changed, the pattern of the relevant factor was comparable. The remaining factors showed comparable associations in the imputed data analysis and the complete data analysis. Moreover, the significant associations in the imputed data were consistent with those in the data when all missing values in maternal work status were imputed as full-time, part-time and unemployed, respectively (data not shown). These sensitivity analyses showed the robustness of results.

### 3.4 Nonresponse analyses

Excluded children (n = 156) were compared to children from included participants (n = 2149) in this study (see Supplementary Table S3). Excluded children were more likely to be a boy and to live with a migrant mother, and to live in a single-parent family (all *p* < 0.05). No significant differences were found in child age and paternal migrant background between these two groups.

## 4 Discussion

This study examined the association between indicators of SES, migration background and psychosocial problems, including social-emotional problems and delay in social-emotional competence, among 2-year-old children. An interaction was observed between maternal migration background and maternal education level. Among children of a mother with a native-born background, a low or middle education level of mothers and fathers was associated with a higher risk of social-emotional problems and a higher risk of competence delay, respectively. For children of a migrant mother, children had a higher risk of both social-emotional problems and competence delay if they had a migrant father, compared to children of a native-born father.

This study adds to the literature by studying the association between SES, parent migration background, and psychosocial problems among 2-year-old children. Existing literature is focused on school-aged children and adolescents (5–18 years)(Kuruczova et al., 2020; Mullola et al., 2021; Kirby et al., 2020). The findings of this study are in line with previous systematic reviews that indicated an increased risk of psychosocial problems in children as socioeconomic deprivation, measured by parent- child- or area level indicators (Poulain et al., 2020). Also, generally a higher risk of both social-emotional problems and competence delay was observed among children of migrant families in this study. This is in line with previous reports of children with a minority background potentially having poor mental health in the Netherlands and Northern European countries (Crone et al., 2010; Mock-Muñoz de Luna et al., 2019). The study underlines the potential impact of the family’s socioeconomic well-being on child’s psychosocial health at preschool age. The first 1000 days of life is a window of opportunity to shape a physical, mental and social healthier future for children (UNICEF, 2017). Early detection and timely intervention to these vulnerable children could not only improve children’s development, but also relieve the burden on society and the health care system due to the high 'rate of return to investment' of investment at a young age (Doyle et al., 2009). The results of our study suggested that for young children living in an adverse SES and/or a migrant family, support may be needed at an early age to promote child’s psychosocial health.

Circa 70% of migrant parents in our study originated from non-western countries. The migration background and corresponding migration, as suggested in previous studies, may partly explain the disparities in children of migrant parents in this study (Kim et al., 2020; Mock-Muñoz de Luna et al., 2019). Moreover, multiple factors may impact these disparities. For example, economic stress within a family could impact children' psychosocial development through parenting: a high level of parenting stress could strengthen the effect of economic disadvantages on children’s internalizing and externalizing problems (Rijlaarsdam et al., 2013). Other suggested factors reported by existing studies include culture/genetic background, ethnic-racial socialization, potential adverse life events, and social/policy-guide attitude in the host country (Crone et al., 2010; Mock-Muñoz de Luna et al., 2019; Wang et al., 2020). In-depth studies, including qualitative research, is needed to gain more insight into pathways to health disparities.

Moreover, in the present study, a significant interaction of maternal education level with maternal migration background was present. Children from mothers with a lower maternal education level had a higher risk of both social-emotional problems and competence delay among children of native-born mothers. It needs to be mentioned that there was a low percentage of children at risk among native-born mothers with a high education level, which could have impacted the findings. However, since education is known to associate with the problem-solving capacity (Liberatos et al., 1988) and the prevalent dominant ethnicity sometimes benefits health advantages (WHO, 2010), this might be reflected in mothers with a high education level and a Dutch background. In the population with migration background, cultural values and practices used could reflect practices from parents’ country of birth (Glick et al., 2012). As such, it has been reported that Turkish and Moroccan migrant fathers might have stricter parenting practices compared to Dutch fathers in the Netherlands (Oosterwegel et al., 2017). In addition, non-Dutch native language might reduce barriers to access professional health care (Kroneman et al., 2016). However, due to the complex mechanism, many individual-, family-, and society factors may impact the association between parental migration background and children’s psychosocial problems, e.g., genetic vulnerability (Gualdi-Russo et al., 2014), parenting environment (Tamura et al., 2020) and cultural socialization (Wang et al., 2020). Therefore, more research including this broad range of family characteristics is needed to confirm our findings and to gain more insight in these associations.

Studies have reported that parental work status has a two-edged sword effect for parents: a positive effect on self-esteem of the parent, but a higher pressure on the life-work balance (Meeussen & Van Laar, 2018; Schober & Schmitt, 2017). Previous findings on the association between parental work status and child’s social-emotional development were contradictory (Arroyo-Borrell et al., 2017; Kirby et al., 2020). In the present study, maternal work status was not associated with children’s higher risk of social-emotional problems or competence delay. This result was in line with a study conducted in the UK regarding the mental health of socioeconomic disadvantaged children at 4–5 years (Kirby et al., 2020). Also, a longitudinal study reported that parental work status in childhood had no significant impact on the onset, persistence, and severity of mental disorders in a US national sample of adults (McLaughlin et al., 2011).

Additionally, living in a one-parent household was not associated with a higher risk of psychosocial problems in this study, which was contrary to previous findings (deKeyser et al., 2014; Herrmann et al., 2018). Single-parent families were reported as a risk factor for child’s development as single parents might lack necessary time and enough income for upbringing (Kulikovskaya et al., 2019). However, both barriers to better parenting could be relieved by sharing the responsibility and maintenance according to the co-parenting policy in the Netherlands (Nikolina, 2015). Future studies are recommended to take work status of both parents as well as living conditions into consideration.

### 4.1 Strengths and limitations

There were three strengths of this study. The first one is the large sample size of 2-year-old children. The second is a broad range of SES indicators. Last one is using an age-specific measurement of psychosocial problems for preschool children—BITSEA. However there are some methodological considerations to be taken into account. Firstly, information regarding family income was unavailable. However, education level has been shown to be a strong indicator of SES(Kuruczova et al., 2020). Secondly, although measured confounders have been selected and taken into account in the regression models, unmeasured confounding cannot be ruled out as previous studies have reported other potential confounding factors, for example, physical/verbal/sexual abuse, physical/emotional neglect, and domestic violence (Pickett et al., 2022). Thirdly, the full models of multivariable logistic regression conducted with non-imputed data and those with imputed data were comparable albeit one factor showed different significance. As most of the factors kept the same direction of the association and significance in both imputed data analysis and non-imputed data analysis, the findings can be considered reliable for both methods. Fourthly, the non-response analyses showed that children and parents included in the current analyses were often higher educated and native-born, amongst others, indicating that our findings are limited to this population. Fifthly, although the BITSEA has been extensively validated (Kruizinga et al., 2012), report bias might be in place. Children may be more likely to have an at-risk score when parents are concerned about their psychosocial health (Briggs-Gowan & Carter, 2008). Moreover, misunderstanding might have existed when migrant parents filled the Dutch version of the BITSEA. Generally, providing the questionnaire in the mother tongue could help improve the vitality of measurement (Crone et al., 2010). Future studies conducted in multiethnic and/or multicultural populations are recommended, using measurements validated in the native language.

## 5 Conclusion

In conclusion, the current study showed that children from families with lower SES as indicated by parental education level and parental migration background, have a higher risk of psychosocial problems at age two years. Health care professionals working with parents and children should be aware of these findings. More studies are needed to evaluate the impact of socioeconomic status and migration background within different family contexts, in relation to child’s psychosocial problems.

## Ethical statement

The Medical Ethical Committee of the Erasmus Medical Center Rotterdam declared that the Medical Research Involving Human Subject Act (Dutch abbreviation WMO) did not apply to the present study and, subsequently, permission was given to carry out the study and to publish the results in scientific journals (number EMC-2014-152). This study was conducted by following the guidelines proposed in the World Medical Association Declaration of Helsinki. Consent forms were obtained from participants. Data used in this study were anonymous on the work platforms.

## Funding statement

This project was supported by 10.13039/501100001826ZonMw (NL) [grant number 729301001]. Jie Luo was funded by the 10.13039/501100010890Chinese Government Scholarship (CN) [grant number 201806170061].

## Author contributions

HR obtained the funding. HR and AG managed the research and undertook data collection. JL, AG, and HR conceived the research described in this paper. JL analyzed the data. All authors provided input in interpreting the data. JL drafted the manuscript with input of AG, HR JY and SJ. SJ checked the writing. All authors critically reviewed and approved the manuscript.

## Declaration of competing interest

The authors declare that they have no conflict of interest.

## Data Availability

Data are not available.
