# Supplementary material for: Psychosocial health disparities in early childhood: Socioeconomic status and parent migration background
Source: SSM Popul Health. 2022 Jun 6;19:101137. doi: 10.1016/j.ssmph.2022.101137 (PMC9194643; doi:10.1016/j.ssmph.2022.101137)
Supplement: Multimedia component 1 [file mmc1.docx]

Supplementary materials

Missing baseline questionnaire

(n=11)

Parents completed the baseline questionnaire at child age 2 years

(n=2305)

Excluded for analyses:

One child in twins (n=31)

Questionnaire completed by other caregiver than parent (n=55)

Missing outcome data (n=70)

Final sample for analyses

(n=2149)

Parents signed the informed consent to participate in the study

(n=2316)

Parents were visited by YHC for their 2-year-old child’s well-child visit

(n=8937)

Supplementary Figure 1. Population of Analysis
