# Supplementary material for: Psychosocial health disparities in early childhood: Socioeconomic status and parent migration background
Source: SSM Popul Health. 2022 Jun 6;19:101137. doi: 10.1016/j.ssmph.2022.101137 (PMC9194643; doi:10.1016/j.ssmph.2022.101137)
Supplement: Multimedia component 2 [file mmc2.docx]

Table 1. Sociodemographic characteristics of the study population at child age 2-years by social-emotional problems and competence delay (N=2149)

|  | Total  (n=2149) |  | Children at risk of social-emotional problems | | *t/* χ2 | *P* value | Effect size |  | Children at risk of competence delay | | *t/* χ2 | *P* value | Effect size |
| --- | --- | --- | --- | --- | --- | --- | --- | --- | --- | --- | --- | --- | --- |
|  |  |  | No  (n=1961) | Yes  (n=188) |  |  |  |  | No  (n=1852) | Yes  (n=297) |  |  |  |
| *Family characteristics* |  |  |  |  |  |  |  |  |  |  |  |  |  |
| Parental age in years | 33.2±5.3 |  | 33.2±5.3 | 32.4±5.7 | 1.97 | 0.050 | 0.16 |  | 33.1±5.2 | 33.8±6.1 | -1.94 | 0.054 | -0.14 |
| Respondent of questionnaire | |  |  |  | 1.11 | 0.293 | 0.02 |  |  |  | 5.41 | **0.020** | 0.05 |
| Mother | 1902 (88.5) |  | 1740 (88.7) | 162 (86.2) |  |  |  |  | 1651 (89.1) | 251 (84.5) |  |  |  |
| Father | 247 (11.5) |  | 221 (11.3) | 26 (13.8) |  |  |  |  | 201 (10.9) | 46 (15.5) |  |  |  |
| Maternal education level |  |  |  |  | 21.44 | **<0.001** | 0.10 |  |  |  | 26.05 | **<0.001** | 0.11 |
| High | 1113 (53.2) |  | 1040 (54.4) * | 73 (40.6)* |  |  |  |  | 1001 (55.4)* | 112 (39.6)* |  |  |  |
| Middle | 796(38.1) |  | 719 (37.6) | 77 (42.8) |  |  |  |  | 662 (36.6)* | 134 (47.3)* |  |  |  |
| Low | 182 (8.7) |  | 152 (8.0)* | 30 (16.7) * |  |  |  |  | 145 (8.0)* | 37 (13.1)* |  |  |  |
| Paternal education level |  |  |  |  | 37.55 | **<0.001** | 0.14 |  |  |  | 10.38 | **0.006** | 0.07 |
| High | 960 (47.5) |  | 915 (49.4)* | 45 (26.6)* |  |  |  |  | 853 (48.7)* | 107 (39.8)* |  |  |  |
| Middle | 788 (39.0) |  | 705 (38.1)* | 83 (49.1)* |  |  |  |  | 676 (38.6) | 112 (41.6) |  |  |  |
| Low | 273 (13.5) |  | 232 (12.5)* | 41 (24.3)* |  |  |  |  | 232 (12.7)* | 50 (18.6)* |  |  |  |
| Maternal work status |  |  |  |  | 21.35 | **<0.001** | 0.11 |  |  |  | 20.22 | **<0.001** | 0.11 |
| Full-time | 261 (14.3) |  | 247 (14.8)* | 14 (9.0)* |  |  |  |  | 228 (14.3) | 33 (14.3) |  |  |  |
| Part-time | 1136 (62.4) |  | 1053 (63.3)* | 83 (53.2)* |  |  |  |  | 1018 (64.1)* | 118 (51.1)* |  |  |  |
| Unemployed | 423 (23.2) |  | 364 (21.9)* | 59 (37.8)* |  |  |  |  | 343 (21.6)* | 80 (34.6)* |  |  |  |
| Maternal migrant background | |  |  |  | 24.97 | **<0.001** | 0.11 |  |  |  | 49.58 | **<0.001** | 0.16 |
| Native-born | 1401 (67.8) |  | 1310 (69.4) | 91 (51.1) |  |  |  |  | 1264 (70.7) | 137 (49.5) |  |  |  |
| Migrant | 664 (32.2) |  | 577 (30.6) | 87 (48.9) |  |  |  |  | 524 (29.3) | 140 (50.5) |  |  |  |
| Paternal migrant background | |  |  |  | 29.89 | **<0.001** | 0.12 |  |  |  | 34.58 | **<0.001** | 0.13 |
| Native-born | 1456 (70.9) |  | 1364 (72.6) | 92 (52.9) |  |  |  |  | 1301 (73.2) | 155 (56.0) |  |  |  |
| Migrant | 598 (29.1) |  | 516 (27.4) | 82 (47.1) |  |  |  |  | 476 (26.8) | 122 (44.0) |  |  |  |
| Family composition |  |  |  |  | 19.28 | **<0.001** | 0.10 |  |  |  | 9.39 | **0.002** | 0.07 |
| Two-parent | 1916 (91.5) |  | 1768 (92.3) | 148 (82.7) |  |  |  |  | 1667 (92.2) | 249 (86.8) |  |  |  |
| Single-parent | 179 (8.5) |  | 148 (7.7) | 31 (17.3) |  |  |  |  | 141 (7.8) | 38 (13.2) |  |  |  |
| *Child characteristics* |  |  |  |  |  |  |  |  |  |  |  |  |  |
| Age in months | 24.6±1.8 |  | 24.6±1.8 | 24.6±1.6 | -0.46 | 0.644 | -0.04 |  | 24.6±1.8 | 24.4±1.8 | 1.92 | 0.055 | 0.12 |
| Gender |  |  |  |  | 13.58 | **<0.001** | 0.08 |  |  |  | 30.65 | **<0.001** | 0.12 |
| Girl | 1072 (49.9) |  | 1002 (51.3) | 70 (37.2) |  |  |  |  | 968 (52.5) | 104 (35.1) |  |  |  |
| Boy | 1069 (50.1) |  | 951 (48.7) | 118 (62.8) |  |  |  |  | 877 (47.5) | 192 (64.9) |  |  |  |
| Previous help seeking |  |  |  |  | 92.77 | **<0.001** | 0.21 |  |  |  | 2.45 | 0.117 | 0.03 |
| No | 1656 (79.5) |  | 1566 (82.1) | 90 (51.4) |  |  |  |  | 1440 (80.1) | 216 (76.1) |  |  |  |
| Yes | 426 (20.5) |  | 341 (17.9) | 85 (48.6) |  |  |  |  | 358 (19.9) | 68 (23.9) |  |  |  |

P values were based on independent T test and χ2 tests. Significant p values are presented in bold.

*Significant difference between two subgroups at 0.05 level in multiple comparison by Bonferroni adjusted z-tests for column proportions. Data presented as mean ± SD or number (percentage).

Number of missing: Parental age=31; Maternal education level=58; Paternal education level=128; Maternal work status=329; Maternal migrant background=84; Paternal migrant background=95; Family composition=54; Child age=15; Child gender=8; Previous help seeking=67.

Table 2. Association between indicators of SES, parental migration background and social-emotional problems (N=2149)

|  | Subgroup of native-born mothers | |  | Subgroup of migrant mothers | |
| --- | --- | --- | --- | --- | --- |
|  | Children at risk of social-emotional problems | Children at risk of competence delay |  | Children at risk of social-emotional problems | Children at risk of competence delay |
|  | OR (95%CI) | OR (95%CI) |  | OR (95%CI) | OR (95%CI) |
| Maternal education level | |  |  |  |  |
| High | Ref | Ref |  | Ref | Ref |
| Middle | 1.24 (0.73-2.12) | 1.86 (1.24-2.78)* |  | 0.56 (0.31-1.01) | 1.42 (0.85-2.36) |
| Low | 1.21 (0.46-3.14) | 1.54 (1.23-5.25)* |  | 1.13 (0.52-2.49) | 1.12 (0.56-2.23) |
| Paternal education level | |  |  |  |  |
| High | Ref | Ref |  | Ref | Ref |
| Middle | 2.38 (1.32-4.31)* | 1.05 (0.69-1.61) |  | 1.83 (0.97-3.43) | 0.92 (0.57-1.49) |
| Low | 3.71 (1.24-5.96)* | 0.71 (0.35-1.47) |  | 1.68 (0.76-3.75) | 1.37 (0.75-2.50) |
| Maternal work status | |  |  |  |  |
| Full-time | Ref | Ref |  | Ref | Ref |
| Part-time | 3.00 (0.89-10.03) | 0.93 (0.51-1.70) |  | 1.51 (0.72-3.19) | 0.97 (0.38-2.40) |
| Unemployed | 3.25 (0.83-12.63) | 1.32 (0.68-2.60) |  | 2.02 (0.97-4.23) | 1.42 (0.75-2.70) |
| Paternal migrant background | |  |  |  |  |
| Native-born | Ref | Ref |  | Ref | Ref |
| Migrant | 1.13 (0.57-2.23) | 1.00 (0.55-1.83) |  | 2.19 (1.21-3.96)* | 1.79 (1.06-3.02)* |
| Family composition | |  |  |  |  |
| Two-parent | Ref | Ref |  | Ref | Ref |
| Single-parent | 1.82 (0.87-3.81) | 1.66 (0.86-3.19) |  | 1.14 (0.63-2.04) | 0.79 (0.46-1.35) |

Abbreviation: OR=odds ratio; CI=confidence internal. The analyses were conducted on imputed data.

The model has been adjusted for covariates: child gender and previous help seeking.

*p<0.05.

Supplementary materials

Missing baseline questionnaire

(n=11)

Parents completed the baseline questionnaire at child age 2 years

(n=2305)

Excluded for analyses:

One child in twins (n=31)

Questionnaire completed by other caregiver than parent (n=55)

Missing outcome data (n=70)

Final sample for analyses

(n=2149)

Parents signed the informed consent to participate in the study

(n=2316)

Parents were visited by YHC for their 2-year-old child’s well-child visit

(n=8937)

Supplementary Figure 1. Population of Analysis

Supplementary Table S1 Maternal and paternal migration background

|  |  | Total  N | At risk of social-emotional problems  N (%) | *P*^1^ | At risk of competence delay  N (%) | *P*^1^ |
| --- | --- | --- | --- | --- | --- | --- |
| Maternal migration background | |  |  |  |  |  |
| Non-western country | | 461 | 70 (15.2)^a^ | 0.017 | 102 (22.1)^b^ | 0.321 |
|  | Morocco | 79 | 7 (8.9) |  | 23 (29.1) |  |
|  | Suriname | 97 | 14 (14.4) |  | 11 (11.3) |  |
|  | Netherlands Antilles/Aruba | 39 | 3 (7.7) |  | 7 (17.9) |  |
|  | Turkey | 67 | 9 (13.4) |  | 17 (25.4) |  |
|  | Indonesia or Moluccas | 54 | 4 (7.4) |  | 11 (20.4) |  |
|  | Cape Verde | 37 | 2 (5.4) |  | 9 (24.3) |  |
|  | Other non-western countries | 142 | 35 (24.6) |  | 35 (24.6) |  |
| Western country | | 203 | 17 (8.4)^a^ |  | 38 (18.7)^b^ |  |
|  | European country | 143 | 12 (8.4) |  | 26 (18.2) |  |
|  | Other western country | 6 | 1 (16.7) |  | 1 (16.7) |  |
| Total |  | 664 | 87 (13.1) |  | 140 (21.1) |  |
| Paternal migration background | |  |  |  |  |  |
| Non-western country | | 422 | 69 (16.4)^c^ | 0.004 | 98 (23.2)^d^ | 0.008 |
|  | Morocco | 85 | 13 (15.3) |  | 23 (27.1) |  |
|  | Suriname | 79 | 13 (16.5) |  | 14 (17.7) |  |
|  | Netherlands Antilles/Aruba | 46 | 3 (6.5) |  | 5 (10.9) |  |
|  | Turkey | 75 | 11 (14.7) |  | 21 (28.0) |  |
|  | Indonesia or Moluccas | 52 | 2 (3.8) |  | 4 (7.7) |  |
|  | Cape Verde | 37 | 4 (10.8) |  | 9 (24.3) |  |
|  | Other non-western countries | 100 | 25 (25.0) |  | 26 (26.0) |  |
| Western country | | 176 | 13 (7.4)^c^ |  | 24 (13.6)^d^ |  |
|  | European country | 120 | 10 (8.3) |  | 19 (15.8) |  |
|  | Other western country | 4 | 1 (25.0) |  | 1 (25.0) |  |
| Total |  | 598 | 82 (13.7) |  | 122 (20.4) |  |

1. Chi-square test was used to compare the proportions marked with the same letter (a, b, c, and d) of children at risk between parents with non-western background and parents with western background.

Supplementary Table S2. Multivariable logistic regression models conducted in complete data (N=2149)

|  | Subgroup of native-born mothers | |  | Subgroup of migrant mothers | |
| --- | --- | --- | --- | --- | --- |
|  | Children at risk of social-emotional problems | Children at risk of competence delay |  | Children at risk of social-emotional problems | Children at risk of competence delay |
|  | OR (95%CI) | OR (95%CI) |  | OR (95%CI) | OR (95%CI) |
| Maternal education level | |  |  |  |  |
| High | Ref | Ref |  | Ref | Ref |
| Middle | 1.09 (0.61-1.98) | 2.38 (1.47-3.85)** |  | 0.59 (0.30-1.17) | 1.05 (0.60-1.85) |
| Low | 1.08 (0.38-3.01) | 2.79 (1.19-6.51)* |  | 0.58 (0.19-1.74) | 0.63 (0.24-1.69) |
| Paternal education level | |  |  |  |  |
| High | Ref | Ref |  | Ref | Ref |
| Middle | 2.63 (1.38-5.01)* | 1.00 (0.62-1.62) |  | 1.52 (0.73-3.17) | 1.04 (0.56-1.91) |
| Low | 2.99 (1.28-7.00)* | 0.59 (0.27-1.29) |  | 1.75 (0.71-4.34) | 1.56 (0.74-3.30) |
| Maternal work status | |  |  |  |  |
| Full-time | Ref | Ref |  | Ref | Ref |
| Part-time | 3.41 (1.00-11.59) | 0.97 (0.49-1.92) |  | 1.66 (0.69-4.02) | 0.92 (0.48-1.77) |
| Unemployed | 3.18 (0.83-12.21) | 1.41 (0.64-3.12) |  | 2.28 (0.96-5.44) | 1.14 (0.59-2.22) |
| Paternal migrant background | |  |  |  |  |
| Native-born | Ref | Ref |  | Ref | Ref |
| Migrant | 1.03 (0.46-2.29) | 1.04 (0.56-1.96) |  | 2.02 (0.98-4.17) | 2.19 (1.20-3.98)* |
| Family composition | |  |  |  |  |
| Two-parent | Ref | Ref |  | Ref | Ref |
| Single-parent | 2.06 (0.79-5.35) | 1.94 (0.83-4.55) |  | 1.66 (0.81-3.39) | 0.78 (0.40-1.52) |

Abbreviation: OR=odds ratio; CI=confidence internal. The analyses were conducted on original data.

Number of missing: Maternal education level=58; Paternal education level=128; Maternal work status=329; Maternal migrant background=84; Paternal migrant background=95; Family composition=54; Child gender=8; Previous care use=67.

The Model is adjusted for covariates: child gender and previous help seeking.

*p<0.05; **p<0.001.

Supplementary Table S3. Non-response analyses (N = 2305)

|  | Total |  | Included in present study | | |  | *p* value |
| --- | --- | --- | --- | --- | --- | --- | --- |
|  | (n=2305)  Mean ± SD N(%) |  | No  (n=156)  Mean ± SD N(%) |  | Yes  (n=2149)  Mean ± SD N(%) |  |  |
| Child age in months | 24.6±1.8 |  | 24.7±1.7 |  | 24.6±1.8 |  | 0.586 |
| Child gender |  |  |  |  |  |  | 0.017 |
| Boy | 1159 (50.6) |  | 90 (60.0) |  | 1069 (49.9) |  |  |
| Girl | 1132 (49.4) |  | 60 (40.0) |  | 1072(50.1) |  |  |
| Maternal migrant background |  |  |  |  |  |  | 0.017 |
| Native-born | 1450 (67.3) |  | 49 (55.7) |  | 1401 (67.8) |  |  |
| Migrant | 703 (32.7) |  | 39 (44.3) |  | 664 (32.2) |  |  |
| Paternal migrant background |  |  |  |  |  |  | 0.064 |
| Native-born | 1507 (70.5) |  | 51 (61.4) |  | 1456 (70.9) |  |  |
| Migrant | 630 (29.5) |  | 32 (38.6) |  | 598 (29.1) |  |  |
| Family composition |  |  |  |  |  |  | <0.001 |
| Two-parent | 2033 (90.9) |  | 117 (83.0) |  | 1916 (91.5) |  |  |
| Single-parent | 203 (9.1) |  | 24 (17.0) |  | 179 (8.5) |  |  |

Note: This table present non-imputed data. The missing numbers of variables are child age (n=21), child gender (n=14), maternal migrant background (n=152), parental migrant background (n=168), family composition (69).

Abbreviation: SD=standard deviation.

P values are based on Independent t-test and chi-square test for non-response to follow-up and response groups.
